# What about the trip? The relationship between ketamine’s acute and antidepressant effect

**DOI:** 10.1192/j.eurpsy.2025.85

**Published:** 2025-08-26

**Authors:** V. Andrashko

**Affiliations:** 1 National Institute of Mental Health, Klecany; 2 Third Faculty of Medicine, Prague, Czech Republic

## Abstract

**Abstract:**

Ketamine has been established as a rapid and potent antidepressant. However, despite its groundbreaking efficacy, it has several limitations, including the transient nature of its antidepressant effects following a single infusion, as well as the phenomenology of acute intoxication. This state is characterized by altered consciousness, manifesting as dissociative or psychotomimetic phenomena, along with cardiovascular changes, such as fluctuations in systolic and diastolic blood pressure.

The presentation explores whether these phenomena should be regarded as mere side effects or if they might play a role in ketamine’s antidepressant mechanisms. Furthermore, it addresses the ongoing challenge of identifying clinical and phenomenological predictors of a better antidepressant response.

**Disclosure of Interest:**

None Declared

